# Modified Shock Index as Simple Clinical Independent Predictor of In-Hospital Mortality in Acute Coronary Syndrome Patients: A Retrospective Cohort Study

**DOI:** 10.3389/fcvm.2022.915881

**Published:** 2022-06-09

**Authors:** Miftah Pramudyo, Vani Marindani, Chaerul Achmad, Iwan Cahyo Santosa Putra

**Affiliations:** Department of Cardiology and Vascular Medicine, Hasan Sadikin General Hospital, Universitas Padjadjaran, Bandung, Indonesia

**Keywords:** modified shock index, global registry of acute coronary events score, acute coronary syndrome, in-hospital mortality, revascularization

## Abstract

**Introduction:**

Despite being the current most accurate risk scoring system for predicting in-hospital mortality for patients with acute coronary syndrome (ACS), the Global Registry of Acute Coronary Events (GRACE) risk score is time consuming due to the requirement for electrocardiography and laboratory examinations. This study is aimed to evaluate the association between modified shock index (MSI), as a simple and convenient index, with in-hospital mortality and revascularization in hospitalized patients with ACS.

**Methods:**

A single-centered, retrospective cohort study, involving 1,393 patients with ACS aged ≥ 18 years old, was conducted between January 2018 and January 2022. Study subjects were allocated into two cohorts: high MSI ≥ 1 (*n* = 423) and low MSI < 1 group (*n* = 970). The outcome was in-hospital mortality and revascularization. The association between MSI score and interest outcomes was evaluated using binary logistic regression analysis. The area under the curve (AUC) between MSI and GRACE score was compared using De Long’s method.

**Results:**

Modified shock index ≥ 1 had 61.1% sensitivity and 73.7% specificity. A high MSI score was significantly and independently associated with in-hospital mortality in patients with ACS [odds ratio (OR) = 2.72(1.6–4.58), *p* < 0.001]. However, ST-segment elevation myocardial infarction (STEMI) and non-STEMI (NSTEMI) patients with high MSI did not significantly increase the probability of revascularization procedures. Receiver operating characteristic (ROC) analysis demonstrated that although MSI and GRACE scores were both good predictors of in-hospital mortality with the AUC values of 0.715 (0.666–0.764) and 0.815 (0.775–0.855), respectively, MSI was still inferior as compared to GRACE scores in predicting mortality risk in patients with ACS (*p* < 0.001).

**Conclusion:**

Modified shock index, particularly with a score ≥ 1, was a useful and simple parameter for predicting in-hospital mortality in patients presenting with ACS.

## Introduction

Acute Coronary Syndrome (ACS) is the most encountered cardiovascular disease manifestation with a high number of morbidity and mortality (1). Until now, ACS has become a disease with the highest mortality rate in developed countries with an expectation to have the same status in developing countries (1). Risk assessment is, therefore, crucial for estimating a patient’s prognosis and indicating the need for a more aggressive approach when needed (2).

Both the Thrombolysis in Myocardial Infarction (TIMI) and the Global Registry of Acute Coronary Events (GRACE) have been widely utilized as ACS risk scoring systems with strong predictive values (2, 3). Compared to TIMI, the GRACE score is more accurate in predicting both short-term and long-term prognoses (4, 5). This score is, arguably, sophisticated as it used tables for calculation and is available through applications and websites (1, 6). However, GRACE score heavily relies on laboratory parameters and electrocardiographic (ECG) findings, hence, time-consuming, and may be difficult to perform routinely bedside (2). A simple and convenient scoring system is, therefore, needed to better assess in-hospital mortality risk in patients with ACS.

Shock index (SI) and modified shock index (MSI) are two emerging and simpler predictors for prognosis in ACS (1). A few studies showed that SI can predict major adverse cardiac events (MACEs) and mortality in ACS (7–9). On the other hand, MSI, which was developed by Ye-cheng et al.,(10, 11) uses the ratio of heart rate (HR) and mean arterial pressure (MAP).

Three observational studies suggested that MSI was useful in predicting mortality for an emergency, medical and trauma patients (11–13). Shangguan et al. suggested that MSI may be more precise than SI in predicting 7-day all-cause mortality and MACEs in ST-segment elevation myocardial infarction (STEMI) patients who underwent emergent percutaneous coronary intervention (PCI) (12). The use of MAP within the calculation of MSI makes its greater predictive power logical as MAP reflects myocardial perfusion and systemic vascular resistance accurately (2). Additionally, unlike SI, the association between MSI and in-hospital mortality in patients with ACS is equivocal. Given the above, this study is aimed to (1) evaluate the impact of MSI on in-hospital mortality and revascularization therapies in patients with ACS prior to and after excluding cardiogenic shock and (2) compare the accuracy of MSI and GRACE scores in predicting in-hospital mortality in patients with ACS.

## Materials and Methods

### Study Design and Patient Selection

This study is a retrospective, single-centered cohort study involving all patients with ACS aged ≥ 18 years old who were hospitalized at Dr. Hasan Sadikin General Hospital, Indonesia between January 2018 and January 2022. The exclusion criteria were missing MSI-related and outcome (in-hospital death or discharge) data from patients’ medical records. This study was approved by the Medical Research Ethics Committee of Dr. Hasan Sadikin General Hospital, West Java, Indonesia. The informed patient consent was collected at the beginning of the study.

### Definition of Variables and Outcome

All data regarding baseline characteristics and diagnosis were collected from patients’ medical records. In this study, ACS was classified into STEMI, non-ST-segment elevation myocardial infarction (NSTEMI), and unstable angina (UA) using criteria established by the European Society of Cardiology guidelines (14, 15). MSI (HR/MAP) and GRACE scores were collected accordingly based on patients’ data in the medical record.

The primary outcome of this study was in-hospital mortality, which is defined as all patients with ACS who died in the hospital before discharge regardless of the cause of death. Additionally, we also evaluated the association between MSI and in-hospital mortality after excluding participants with cardiogenic shock. The secondary outcome was a revascularization procedure that includes fibrinolytic and PCI in patients with STEMI and NSTEMI prior to and after excluding cardiogenic shock.

### Statistical Analysis

All statistical analyses were completed using SPSS version 25.0 (IBM Corp., Armonk, NY, United States) and MedCalc software for Windows, version 20.106 (MedCalc Software, Mariakerke, Belgium). Data distribution was evaluated using the Kolmogorov-Smirnov test. Numerical variables with parametric distributions were presented as mean ± standard deviation (SD), while numerical variables with non-parametric distributions were presented as median and interquartile range. Total numbers and percentages were reported for categorical variables. A Mann–Whitney *U* test was used to evaluate the differences between two numerical variables. To compare the differences between two categorical variables, we performed the chi-square test or Fisher’s exact test, as indicated. The association between MSI score and the outcome of interest was evaluated using binary logistic regression analysis with the backward method. The cut-off of MSI was determined by receiver operating characteristics (ROC) analysis. Multivariate analysis was performed by adjusting several confounding factors with a *p*-value < 0.25 based on the univariate analysis. Statistical results were presented as an odds ratio (OR) with a 95% confidence interval (CI) and a *p*-value. We used a two-tailed *p*-value with a significance set at ≤ 0.05. ROC analysis was also performed to assess the accuracy of MSI and GRACE risk scores in predicting in-hospital mortality. The area under the curve (AUC) between two scoring systems was compared using De Long’s method.

## Results

The optimal cut-off for MSI was chosen according to ROC curve analysis. This analysis revealed that the optimal cut-off for MSI was one based on a value that had the closest distance to the upper left corner of the ROC curve. The sensitivity and specificity were 61.1 and 73.7%, respectively. Patients were divided into two groups including the high MSI group with MSI being equal to and higher than one (n = 423) and the low MSI group with MSI being lower than one (*n* = 970).

### Baseline Characteristics

**FIGURE 1:**
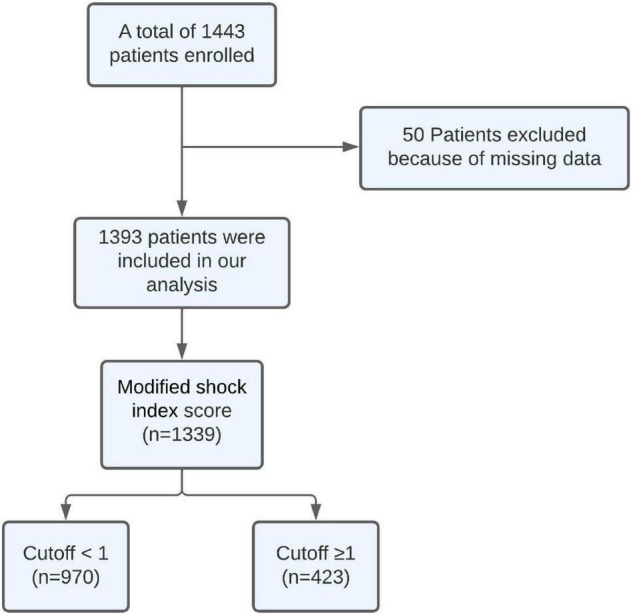
Participants’ selection process.

A total of 1,443 patients with ACS were hospitalized at Dr. Hasan Sadikin General Hospital between January 2018 and January 2022. Fifty participants were excluded because of missing data. Hence, a total of 1,393 participants were included and analyzed in this study. All included participants completed the follow-up period in the hospital with a median follow-up duration being 4 (2.5–5.5) days. The patient selection process is described in Figure 1.

Baseline characteristics are described in Table 1. Patients with high MSI were older (*p* = 0.008) and had a lower incidence of hypertension (54.1 vs. 66.6, *p* < 0.001) and dyslipidemia (14.7 vs. 20.7, *p* = 0.008) when compared to low MSI. Patients with high MSI had a longer duration of chest pain prior to admission as compared to patients with low MSI (*p* < 0.001). Patients with STEMI are more likely to present with high MSI (66.2 vs. 54.6, *p* < 0.001). While patients with NSTEMI (30 vs. 38, *p* = 0.004) and UA (3.8 vs. 6.8, *p* = 0.028) apparently have lower MSI. Patients with high MSI had significantly higher BMI (*p* = 0.004). Regarding laboratory results at admission, patients with high MSI had significantly higher blood glucose (*p* < 0.001), serum urea (*p* < 0.001), leukocyte (*p* < 0.001), creatinine (*p* < 0.001), and troponin (*p* < 0.001) levels. Lastly, patients with high MSI were less possibly to receive primary PCI as compared to those with lower MSI scores (57.3 vs. 65.8, *p* = 0.003).

**TABLE 1:** Baseline characteristics.

Variable	Modified shock index score		*p*-value
	
	≥ 1 (*n* = 423)	< 1 (*n* = 970)	
**Demographic and lifestyle**			
Age (years), median (IQR)	59 (44–74)	57 (42–72)	0.008
Male, *n* (%)	325 (76.8)	741 (76.4)	0.801
**Smoking status**			
Current, *n* (%)	240 (56.7)	580 (59.8)	0.288
**Risk factors**			
Hypertension, *n* (%)	229 (54.1)	646 (66.6)	< 0.001
Type II DM, *n* (%)	100 (23.6)	199 (20.5)	0.190
Dyslipidemia, *n* (%)	62 (14.7)	201 (20.7)	0.008
Family history of premature CAD, *n* (%)	41 (9.7)	98 (10.1)	0.825
Obesity, *n* (%)	110 (26)	317 (32.7)	0.013
Chest pain duration prior admission (hours)	14 (1–27)	10 (1–19)	< 0.001
Chest pain > 12 h	245 (57.9)	441 (45.5)	< 0.001
**Killip classification**			
Killip II, *n* (%)	95 (22.5)	126 (13)	< 0.001
Killip III, *n* (%)	19 (4.5)	20 (2.1)	0.011
Killip IV, *n* (%)	101 (23.9)	38 (3.9)	< 0.001
**ACS types**			
STEMI, *n* (%)	280 (66.2)	530 (54.6)	< 0.001
NSTEMI, *n* (%)	127 (30)	369 (38)	0.004
UAP, *n* (%)	16 (3.8)	71 (7.3)	0.028
SBP (mmHg), median (IQR)	105 (76–134)	130 (100–160)	< 0.001
DBP (mmHg), median (IQR)	70 (50–90)	80 (60–100)	< 0.001
MAP (mmHg), median (IQR)	83.3 (65.3–101.3)	95.5 (73.4–117.6)	< 0.001
Heart rate (bpm), median (IQR)	98 (78–118)	74 (55–93)	< 0.001
**Laboratory findings at admission**			
Direct blood glucose (mg/dL)	135.5 (74.5–196.5)	127 (69–185)	< 0.001
Hemoglobin (g/dL), median (IQR)	14.25 (10.35–18.15)	14.2 (10.7–14.2)	0.563
Leukocyte (10^9^/L), median (IQR)	12,515 (5,897–19,133)	10,930 (6,087–15,773)	< 0.001
Ureum (mmol/L), median (IQR)	46.2 (10–82.4)	33 (6.6–59.4)	< 0.001
Creatinine (μmol/L), median (IQR)	1.5 (0.4–2.6)	1.19 (0.29–1.48)	< 0.001
Troponin-I (ng/L), median (IQR)	10 (2.05–17.95)	6.15 (0.01–12.29)	< 0.001
**Revascularication procedure**			
Fibrinolytic, *n* (%)	47 (11.1)	111 (11.4)	0.711
PCI, *n* (%)	243 (57.4)	638 (65.8)	0.003

*All numerical variables were presented in median (interquartile range) and SI units. All categorical variables were presented in n (%). IQR, interquartile range; DM, diabetes mellitus; MI, myocardial infarction; SBP, systolic blood pressure; DBP, diastolic blood pressure; MAP, mean arterial pressure; PCI, primary coronary intervention.*

### The Association Between MSI Scores and Primary Outcomes

The total in-hospital mortality calculated for all participants was 162 (12.1%). Univariate analysis indicated that a high MSI (≥ 1) was significantly associated with in-hospital mortality in patients with ACS as compared to a low MSI (< 1) before [OR = 4.36 (3.09–6.14), *p* < 0.001] and after excluding participants with cardiogenic shock [OR = 2.88 (1.89–4.38), *p* < 0.001]. Furthermore, multivariate analysis was completed by adjusting several confounding factors with *p* < 0.25 in the univariate analysis including age, sex, smoke, diabetes mellitus (DM) type II, hypertension, obesity, chest pain duration > 12 h, Killip II–IV, blood glucose level, hemoglobin, leukocyte, troponin level, fibrinolytic, and PCI (Table 1). Multivariate analysis showed that high MSI (≥ 1) was significantly associated with in-hospital mortality in patients with ACS before [OR = 2.64 (1.67–4.20), *p* < 0.001] and after excluding cardiogenic shock [OR = 2.68 (1.57–4.55), *p* < 0.001]. The results of the univariate and multivariate analyses are described in Tables 2, 3.

**TABLE 2:** Univariate and multivariate analysis of association between several factors and in-hospital mortality in patients with ACS.

Variables	Crude OR (95% CI)	*p-v*alue	Adjusted OR (95% CI)	*p-v*alue
Age	1.06 (1.04–1.07)	< 0.001	1.05 (1.03–1.07)	< 0.001
Male	0.57 (0.40–0.81)	0.002	0.67 (0.39–1.13)	0.133
Current smoker	0.64 (0.46–0.88)	0.007	1.15 (0.68–1.92)	0.609
Hypertension	1.29 (0.91–1.83)	0.160	1.22 (0.76–1.94)	0.408
Type II DM	1.46 (1.01–2.13)	0.044	0.89 (0.52–1.53)	0.664
Dyslipidemia	0.96 (0.63–1.47)	0.843		
Obesity	0.74 (0.50–1.07)	0.110	0.70 (0.43–1.15)	0.162
Family history of premature CAD	0.93 (0.53–1.64)	0.805		
Chest pain > 12 h	1.48 (1.06–2.07)	0.021	0.98 (0.63–1.52)	0.924
Killip II-IV	5.65 (3.98–8.01)	< 0.001	3.99 (2.58–6.18)	< 0.001
Direct blood glucose (mg/dL)	1.004 (1.002–1.005)	< 0.001	1.003 (1.000–1.005)	0.032
Hemoglobin (g/dL)	1.00 (0.99–1.00)	0.123	1.00 (0.99–1.00)	0.346
Leukocyte (10^9^/L)	1.00 (1.00–1.00)	< 0.001	1.00 (1.00–1.00)	< 0.001
Ureum (mmol/L)	1.00 (1.00–1.00)	0.567		
Creatinine (μmol/L)	1.001 (0.99–1.003)	0.376		
Troponin-I (ng/L)	0.998 (0.995–1.001)	0.179	0.999 (0.995–1.002)	0.510
Fibrinolytic	0.41 (0.21–0.83)	0.011	0.59 (0.28–1.26)	0.174
PCI	0.36 (0.26–0.51)	< 0.001	0.40 (0.26–0.61)	< 0.001

*All numerical variables were presented in median (interquartile range) and SI units. All categorical variables were presented in n (%). DM, diabetes mellitus; PCI, primary coronary intervention.*

**TABLE 3:** Univariate and multivariate analysis of association between high modified shock index (≥ 1) and in-hospital mortality in patients with ACS.

Variables	Crude OR (95% CI)	*p*-value	Adjusted OR (95% CI)	*p*-value
**Prior excluding cardiogenic shock (*n* = 1,393)**				
In-hospital mortality	4.36 (3.09–6.14)	< 0.001	2.64 (1.67–4.20)	< 0.001
**After excluding cardiogenic shock (*n* = 1,254)**				
In-hospital mortality	2.88 (1.89–4.38)	< 0.001	2.68 (1.57–4.55)	< 0.001

*OR, odds ratio.*

### The Association Between MSI Scores and Secondary Outcomes

A high MSI score was significantly associated with lower PCI procedure in STEMI [OR = 0.69 (0.51–0.94), *p* < 0.001] and NSTEMI populations [OR = 0.54 (0.36–0.80), *p* = 0.002] on univariate analysis. After excluding cardiogenic shock, the significant association was only obtained in the NSTEMI population [OR = 0.63 (0.41–0.97), *p* = 0.035].

Multivariate analysis was performed by adjusting several confounding factors including age, DM type II, family history of premature cardiovascular disease (CVD), chest pain duration prior to admission, Killip classification, blood glucose level, hemoglobin, leukocyte, and troponin level. It showed that high MSI was independently associated with lower PCI procedure in the NSTEMI population before [OR = 0.54 (0.32–0.91), *p* = 0.022] and after excluding cardiogenic shock [OR = 0.55 (0.32–0.95), *p* = 0.03].

On the other hand, univariate and multivariate analyses found no significant association between high MSI and fibrinolytic use in the STEMI population prior to or after excluding cardiogenic shock. Univariate and multivariate analyses of the association between high MSI and revascularization therapies are described in Table 4.

**TABLE 4:** Univariate and multivariate analysis of association between high modified shock index (≥1) and revascularization therapies in patients with ACS.

Variables	Crude OR (95% CI)	*p-v*alue	Adjusted OR (95% CI)	*p-v*alue
**Prior excluding cardiogenic shock**				
**STEMI participants (*n* = 810)**				
Fibrinolytic	0.77 (0.53–1.14)	0.189	0.90 (0.56–1.44)	0.655
PCI	0.69 (0.51–0.94)	0.020	0.79 (0.56–1.14)	0.205
**NSTEMI participants (*n* = 496)**				
PCI	0.54 (0.36–0.80)	0.002	0.50 (0.32–0.78)	0.002
**After excluding cardiogenic shock**				
**STEMI participants (*n* = 702)**				
Fibrinolytic	0.84 (0.55–1.29)	0.43	0.87 (0.54–1.42)	0.587
PCI	0.74 (0.52–1.06)	0.101	0.88 (0.60–1.31)	0.536
**NSTEMI participants (*n* = 466)**				
PCI	0.63 (0.41–0.97)	0.035	0.61 (0.38–0.97)	0.039

*STEMI, ST-segment elevation myocardial infarction; NSTEMI, non-ST segment elevation myocardial infarction; PCI, percutaneous coronary intervention; OR, odds ratio.*

### ROC Analysis of MSI and GRACE Risk Scores in Predicting In-Hospital Mortality in Patients With ACS

**FIGURE 2:**
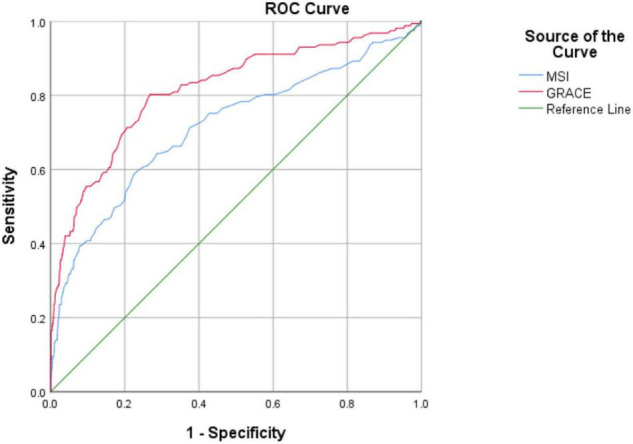
Receiver operating characteristic analysis.

In the ACS population, ROC analysis demonstrated that MSI and GRACE risk scores were a good predictor of in-hospital mortality with the AUC of 0.715 (0.666–0.764) and 0.815 (0.775–0.855), respectively. This analysis showed that the predictive accuracy of MSI scores was significantly lower than the GRACE risk score (*p* < 0.001). ROC analyses are described in Figure 2.

## Discussion

To the best of our knowledge, this is the first cohort study that evaluated the association between MSI and in-hospital mortality in the overall ACS population. Following are the key findings of this study: first, patients with ACS presenting with high MSI (≥1) at admission had a significantly and independently higher risk for in-hospital mortality as compared to those with lower MSI (<1) regardless of cardiogenic shock status. Second, interestingly, NSTEMI patients with high MSI did not increase yet significantly and independently reduced the probability of revascularization management as opposed to patients with low MSI. Whereas STEMI patients with a high MSI were not associated with revascularization therapies in contrast to patients with low MSI. Third, although MSI and GRACE risk scores were good predictors of in-hospital mortality, the predictive accuracy of MSI scores was significantly lower than the GRACE risk score. Thus, our study showed that MSI is a fast and simple indicator for predicting in-hospital mortality outcomes in patients with ACS.

Modified shock index is a readily available index, which is independent of subjective information (e.g., previous patient history) and ECG findings or blood tests; it only depends on the measurement of blood pressure (BP) and HR at admission. Previously, MSI is a widely known valid prognostic tool to predict mortality risk in medical or trauma patients in the emergency room (11, 13, 16). Nonetheless, it is reasonable to use MSI as a predictor of mortality in patients with ACS due to each of its components associated with mortality risk in patients with ACS. An observational study conducted by Shiraishi et al. involving 1,413 patients with primary PCI reported that low MAP at admission (MAP < 79 mmHg) might be associated with a higher in-hospital mortality rate (17). Furthermore, an observational study by Dobre et al. consisting of 22,398 patients with AMI and heart failure showed that a higher HR was independently associated with all-cause mortality and cardiovascular mortality (18).

Several explanations of the pathophysiology of the association between MSI and mortality in patients with ACS have been proposed (7). First, MAP, a derivate of MSI, may indicate any deterioration in left ventricular (LV) stroke function, cardiac stroke volume, and cardiac index (19). Hence, MAP reduction reflecting high MSI is indicative of serious cardiac dysfunction and eventually, extensive left ventricular remodeling and heart failure, in which, the latter comes with a higher mortality rate (7). Second, HR increment presented by higher MSI might demonstrate the sympathetic nervous system overactivity. Patients with ACS mostly experience an overactivity of the sympathetic nervous system, leading to a higher degree of LV dysfunction (20). It is also associated with fatal ventricular arrhythmias, which is a prevalent complication leading to a large proportion of sudden cardiac death (21). In addition, MSI is a relatively an objective indicator of disease state (10) as it is independent, mostly, of pain and anxiety (22), which is a cause of an increase in HR and BP. Thus, combining MAP and HR into one index may represent the true hemodynamic status and end-organ perfusion in patients with ACS.

In our study, patients with high MSI were older (*p* = 0.008), which is consistent with Gouda et al.’s study findings (*p* < 0.001) (10). However, patients with high MSI had a lower incidence of prior hypertension, dyslipidemia, and obesity. While smoking status, DM type II, and family history of premature CAD were not different between the two MSI groups. Consistently, several cohort studies also revealed an insignificant difference in patients’ comorbidities between both MSI groups (2, 10, 12). Thus, it concludes that patients’ comorbidities were not associated with increased MSI.

Regarding clinical presentation at admission, patients with high MSI were more likely to get Killip II–IV presentation and had higher HR (*p* < 0.001), lower diastolic BP (DBP; *p* < 0.001), lower systolic BP (SBP; *p* < 0.001), and lower MAP (< 0.001). Consistently, our results were similar to Gouda et al.’s study showing that patients with high MSI scores were more likely to present with Killip class II–IV (*p* = 0.014) and had lower SBP and DBP (all *p* < 0.001) (10). Furthermore, our study showed that increased MSI was associated with higher blood glucose, leukocytes, urea, creatinine, and troponin levels as compared to low MSI. Thus, it concludes that increased MSI was more likely to appear in critical and morbid patients with ACS.

In our study, increased MSI was significantly associated with an increased risk of in-hospital mortality among patients with ACS. Consistently, three cohort studies had similar results to ours. A prospective cohort study conducted by Gouda et al. found that a higher MSI score was related to an increase in cardiogenic shock, fatal arrhythmia, bleeding, arrest, and mortality incidence. The optimal cut-off value of MSI for predicting in-hospital all-cause mortality and MACE in patients with STEMI was 0.91 with sensitivity and specificity values of 80.0 and 56.2%, respectively (10). Moreover, a retrospective cohort study by Shangguan et al. showed that high MSI (> 1.4) significantly increased the incidence of MACE, fatal arrhythmia, all-cause mortality, and higher Killip class within 7 days (12). More recent retrospective cohort study by Abreu et al. enrolled 1,140 STEMI patients, including those who received either both pharmacological or mechanical reperfusion and suggested that high MSI (>1.3) significantly increased the incidence of malignant arrhythmia, mechanical complications, and respiratory tract infections during hospitalization, and in-hospital mortality (2). Lastly, although MSI may be a more practical index, it was inferior as compared to the GRACE score in predicting the risk of in-hospital mortality in patients with ACS.

Surprisingly, this cohort revealed that high MSI group was significantly and independently reduced the probability of PCI procedures in NSTEMI patients compared to the low MSI group. On the other hand, STEMI patients presented with high MSI was not significantly and independently linked with revascularization procedures (PCI and fibrinolytic). The possible explanation of these phenomena is because there are several factors that affect the clinician’s decision in performing revascularization procedures including chest pain duration prior to admission, patients’ risk profile for NSTEMI, patients with fibrinolytic-related contraindication (e.g., high risk of bleeding), and other patient-related factors (e.g., patient preference, vulnerability, functional, and mental status). Consistently, this study revealed that patients with high MSI were presented with a longer duration of chest pain as compared to patients with low MSI. However, other factors that affect the revascularization procedures except for the chest pain duration were not collected in this study.

Despite its promising results, several limitations should be addressed. First, it was a single-centered, retrospective, observational study, which might impose selection bias, and thus confounders. Second, the authors cannot control the patients’ medication, which might influence their HR and/or BP at admissions, such as antihypertensive, beta-blockers, inotropes, and vasopressors. Third, HR and BP were taken at only a single time point, which was undoubtedly different from those taken within a subsequent measurement, as some adjustments might alter HR and BP values, which were not estimated by ambulatory monitoring. Fourth, this study did not investigate other possible outcomes and the long-term mortality risks. Fifth, other causes of in-hospital mortality except ACS were not further analyzed in this study because of lack of data, resulting in an increased risk of bias. Sixth, NSTEMI patients’ risk profile was not collected in this study, it may cause bias in the association between high MSI and PCI procedure in patients with NSTEMI. Lastly prospective, multi-centered cohort studies with larger sample sizes, indicators collection in different periods, more additional outcomes, and longer follow-up duration are highly required to evaluate the association between MSI and deleterious outcomes in the ACS population better.

## Conclusion

In conclusion, our retrospective cohort study showed that high MSI (≥ 1) was significantly and independently associated with in-hospital mortality in patients with ACS. Moreover, possibly due to several factors, such as chest pain duration prior to admission and other patients’ related factors, revascularization procedures were not significantly higher in STEMI and NSTEMI patients with high MSI (≥ 1). However, despite being more practical and convenient, the MSI score has a lower predictive value when compared to the GRACE score.

## Data Availability Statement

The raw data supporting the conclusions of this article will be made available by the authors, without undue reservation.

## Ethics Statement

The studies involving human participants were reviewed and approved by Health Research Ethics Committee, Faculty of Medicine, Padjadjaran university. The patients/participants provided their written informed consent to participate in this study.

## Author Contributions

MP: conceptualization, methodology, investigation, and writing – original draft, review, and editing. VM: conceptualization, methodology, data curation, and writing – original draft. CA: conceptualization, and writing – original draft, review, and editing. IP: methodology, formal analysis, investigation,and writing – original draft.

## Conflict of Interest

The authors declare that the research was conducted in the absence of any commercial or financial relationships that could be construed as a potential conflict of interest.

## Publisher’s Note

All claims expressed in this article are solely those of the authors and do not necessarily represent those of their affiliated organizations, or those of the publisher, the editors and the reviewers. Any product that may be evaluated in this article, or claim that may be made by its manufacturer, is not guaranteed or endorsed by the publisher.
